# Numerical Generation of Trajectories Statistically Consistent with Stochastic Differential Equations

**DOI:** 10.3390/e27070729

**Published:** 2025-07-06

**Authors:** Mykhaylo Evstigneev

**Affiliations:** Department of Physics and Physical Oceanography, Memorial University of Newfoundland, St. John’s, NL A1B 3X7, Canada; mevstigneev@mun.ca

**Keywords:** Langevin equation, Fokker–Planck equation, multiplicative noise, computational physics, cumulants

## Abstract

A weak second-order numerical method for generating trajectories based on stochastic differential equations (SDE) is developed. The proposed approach bypasses direct noise realization by updating the system’s state using independent Gaussian random variables so as to reproduce the first three cumulants of the state variable at each time step to the second order in the time-step size. The update rule for the state variable is derived based on the system’s Fokker–Planck equation in an arbitrary number of dimensions. The high accuracy of the method as compared to the standard Milstein algorithm is demonstrated on the example of Büttiker’s ratchet. While the method is second-order accurate in the time step, it can be extended to systematically generate higher-order terms of the stochastic Taylor expansion approximating the solution of the SDE.

## 1. Introduction

Stochastic differential equations (SDEs) are an essential tool for research and modelling across a wide range of disciplines, from physics to biology, climatology, and finance [1,2,3]. The standard schemes to solve a SDE are based on the stochastic Taylor expansion [4,5,6,7,8,9] illustrated here on a one-dimensional Langevin equation (LE):

(1) $$\dot{z}\left(t\right)=h\left(z\left(t\right)\right)+g\left(z\left(t\right)\right)\;\xi \left(t\right)\;,$$

in which h(z) and g(z) are known functions of the state variable *z*, the overdot indicates the time derivative, and the noise ξ(t) is a random function of time. To derive the update rule for the state variable from the current time *t* to the next time t+Δt, an integration of Equation (1) is first performed:

(2) $$z\left(t^{\prime }\right)=z\left(t\right)+\int_{t}^{t^{\prime }}ds\;\left(h(z(s))+g(z(s))\;\xi (s)\right)\;,$$

over an interval $t<t^{\prime }\leq t+\Delta t$ not exceeding the time step Δt intended to be used in the numerical solution of (1). Within the integral (2), the functions h(z(s)) and g(z(s)) are Taylor-expanded around the initial point z(t) to a desired order, and the original Equation (2) is then back-substituted into this expansion. Finally, the state variable z(s) in the integral is replaced with its initial value z(t), and the upper integration limit is set to $t^{\prime }=t+\Delta t$. As a result, one obtains the next-step value $z^{\prime }=z\left(t+\Delta t\right)$ in terms of the initial value z=z(t). In the lowest order, one recovers the Milstein algorithm [10], a standard method of solving SDEs, whose various extensions and refinements exist [8,11,12,13,14,15].

The so-obtained numerical scheme is a strong one, i.e., it reproduces the stochastic trajectory for each specific noise function ξ(t). For most practical purposes, however, the explicit stochastic trajectory z(t) corresponding to a particular noise realization ξ(t) is usually unnecessary. Instead, one typically aims to generate multiple trajectories that are statistically consistent with the SDE, allowing for the computation of meaningful quantities, such as average values and correlation functions of various types. This statistical approach, also known as a weak integration scheme of the SDE (1), is equally valuable as the strong one.

Building upon this perspective, alternative methods were introduced [16,17], in which the system’s state at the next time step t+Δt is treated as a random variable sampled from the conditional probability density $P(z^{\prime },t+\Delta t|z,t)$ at time t+Δt given the initial state at time *t*. Due to the smallness of Δt, efficient approximations for the function $P(z^{\prime },t+\Delta t|z,t)$ can be developed.

As a matter of fact, the full conditional probability density is not necessary for the generation of stochastic trajectories; knowing only its first few moments suffices to develop an effective numerical procedure for trajectory generation [18]. This principle was applied in a recent study [19], where the Langevin Equation (1) driven by Gaussian white noise ξ(t) was analyzed for a one-dimensional system.

The present work generalizes the weak second-order scheme from [19] to the white noise-driven LE in arbitrary dimensionality *N*. The significance of the method developed here lies in its capacity to systematically generate the terms in the weakly convergent stochastic Taylor expansion. In this respect, it plays a role analogous to the stochastic Taylor expansion formulated by Kloeden and Platen for strongly convergent schemes [4]. Notably, the Milstein algorithm emerges as the first-order truncation of either expansion in Δt: the strong Taylor expansion of Kloeden–Platen or the weak one presented in this work. The formulation developed here can serve as a benchmark framework for assessing the accuracy of integration schemes not explicitly derived from stochastic Taylor expansions, such as midpoint-type algorithms, leapfrog methods, predictor–corrector schemes, or stochastic Runge–Kutta methods.

This paper is structured as follows. In the next section, the main result of this work is formulated, which is a recipe to generate the system’s state $z_{n}^{\prime }$ at the time t+Δt given the initial state $z_{n}$ at the earlier simulation time step *t* for a multidimensional version of the LE (1) with Gaussian white noises. The subsequent section focuses on the derivation of this update rule. An illustrative example, Büttiker ratchet driven by a spatially periodic temperature profile, is then provided and analyzed using the method developed in this paper and with the help of the classical Milstein scheme. Finally, a possible systematic improvement of the method is briefly discussed and a few concluding remarks are made.

## 2. Numerical Generation of Stochastic Trajectories

Consider a system whose state, represented by a collection of variables *z* = $(z_{1}$, $z_{2}$, $\ldots ,z_{N})$, evolves in time according to the *N*-dimensional LE:

(3) $$\begin{array}{c} {\dot{z}}_{i}=h_{i}\left(z\right)+g_{ij}\left(z\right)\;\xi_{j}\left(t\right)\;,\\ \langle \xi_{i}\left(t\right)\rangle =0\;,\;\;\langle \xi_{i}\left(t\right)\;\xi_{j}\left(t^{\prime }\right)\rangle =\delta_{ij}\;\delta \left(t-t^{\prime }\right)\;, \end{array}$$

in which the functions $h_{i}\left(z\right)$ and $g_{ij}\left(z\right)$ are real-valued and differentiable at least three times, the matrix $g_{ij}\left(z\right)$ is non-degenerate for all values of the state variable *z*, the independent noises $\xi_{i}\left(t\right)$ are unbiased, Gaussian, and white, the noise term $g_{ij}\left(z\right)\;\xi_{j}\left(t\right)$ is interpreted in the Stratonovich sense, and the summation is implied for repeated indices.

Suppose that the system was in a state z=z(t) at time *t* and entered a state

(4) $$z^{\prime }=z\left(t+\Delta t\right)=z+\Delta z$$

at the next time t+Δt. The central result of this paper is the propagation rule $z\to z^{\prime }$:

(5) $$z_{n}^{\prime }=z_{n}+a_{n}+b_{nm}\;\Gamma_{m}+c_{nml}\left(\Gamma_{m}\Gamma_{l}-\delta_{ml}\right)$$

where $\Gamma_{n}$ are independent Gaussian random variables drawn from the probability density

(6) $$W\left(\Gamma_{1},\Gamma_{2},\ldots \right)=\prod_{i}\frac{e^{-\Gamma_{i}^{2}/2}}{\sqrt{2\pi }}\;,$$

and the coefficients are given by

(7) $$\begin{array}{cc} \; & a_{n}=\Delta t\;H_{n}+\frac{\Delta t^{2}}{2}\;\left(G_{ij}\;H_{n,i,j}+H_{i}\;H_{n,i}\right)+O\left(\Delta t^{3}\right)\;,\\ \; & b_{nm}=g_{nm}\;\sqrt{\Delta t}+\Delta t^{3/2}\;\left(\frac{1}{2}\;\left(H_{n,i}g_{im}+G_{ij}g_{nm,i,j}+H_{i}g_{nm,i}\right)+\sigma_{n[ij]}\;\sigma_{k[ij]}\;{\left(g^{-1}\right)}_{mk}\right)\\ \; & \;\;\;\;\;\;\;+O(\Delta t^{5/2})\;,\\ \; & c_{nml}=\Delta t\;\sigma_{nml}+O\left(\Delta t^{2}\right)\;. \end{array}$$

For notational brevity the argument *z* is suppressed in the right-hand sides, where $g_{ij}\equiv g_{ij}\left(z\right)$ and

(8) $$\begin{array}{c} G_{ij}=\frac{1}{2}g_{ik}g_{jk}\;,\;\;H_{i}=h_{i}+\sigma_{ijj}\;,\;\;\sigma_{ijk}=\frac{1}{2}\;g_{ij,l}g_{lk} \end{array}$$

are likewise functions of the initial state *z*. Partial differentiation is represented by a comma, e.g., $\partial A/\partial z_{i}\equiv A_{,i}$, and square brackets around subscripts indicate antisymmetrization, whereas the round brackets will signify symmetrization. Namely, for any object $A_{i_{1}\ldots i_{k}}$ that depends on *k* indices $i_{1},\ldots ,i_{k}$, its symmetrized and antisymmetrized versions are

(9) $$A_{\left(i_{1}\ldots i_{k}\right)}=\frac{1}{k!}\sum_{P}A_{P(i_{1}\ldots i_{k})}\;\;,\;\;\;A_{\left[i_{1}\ldots i_{k}\right]}=\frac{1}{k!}\sum_{P}{\left(-1\right)}^{P}A_{P(i_{1}\ldots i_{k})}\;,$$

where summation is performed over all permutations P of the subscripts $i_{1},\ldots ,i_{k}$, and ${\left(-1\right)}^{P}$ equals 1 or −1 for even and odd permutations, respectively. We, furthermore, adopt the convention that the dummy indices inside the brackets are not to be symmetrized over; hence, placement of the brackets is important. For example,

(10) $$A_{\left(ijkk\right)}\equiv A_{(ij)kk}=\frac{1}{2}\left(A_{ijkk}+A_{jikk}\right)$$

is, in general, not equal to

(11) $$A_{(ijk)k}=\frac{1}{6}\left(A_{ijkk}+A_{jikk}+A_{ikjk}+A_{jkik}+A_{kijk}+A_{kjik}\right)\;,$$

unless $A_{ijkl}$ is symmetric in the first three subscripts.

## 3. Derivation of the Propagation Rule

The idea of the derivation is to choose the coefficients $a_{n}$, $b_{nm}$, and $c_{nml}$ in Equation (5) so as to correctly reproduce the first three moments $\mu_{n}=\bar{\Delta z_{n}}$, $\mu_{nm}=\bar{\Delta z_{n}\;\Delta z_{m}}$, and $\mu_{nml}=\bar{\Delta z_{n}\;\Delta z_{m}\;\Delta z_{l}}$ of the displacements $\Delta z_{n}=z_{n}^{\prime }-z_{n}$. The moments can be obtained from the characteristic function

(12) $$C\left(\zeta \right)=\bar{e^{\zeta_{s}\;\Delta z_{s}}}=e^{-\zeta_{s}z_{s}}\;\bar{e^{\zeta_{s}z_{s}^{\prime }}}$$

by differentiation, e.g., $\mu_{n}=\partial C/\partial \zeta_{n}{|}_{\zeta =0}$, etc. To ensure the existence of C(ζ), the parameters $\zeta_{n}$ are purely imaginary.

Alternatively, we may focus on the first three cumulants, defined as the derivatives of the characteristic function’s natural logarithm [2],

(13) $$\kappa_{n_{1}\ldots n_{k}}=\frac{\partial \ln C(\zeta )}{\partial \zeta_{n_{1}}\ldots \partial \zeta_{n_{k}}}{|}_{\zeta =0}\;.$$

The first three cumulants happen to coincide with the respective central moments and are expressed in terms of the unknown coefficients in Equation (5) as

(14) $$\begin{array}{c} \kappa_{n}=\bar{\Delta z_{n}}=a_{n}\;,\\ \kappa_{nm}=\bar{\left(\Delta z_{n}-\bar{\Delta z_{n}}\right)\;\left(\Delta z_{m}-\bar{\Delta z_{m}}\right)}=b_{ni}\;b_{mi}+2\;c_{n(ij)}\;c_{m(ij)}\;,\\ \kappa_{nml}=\bar{\left(\Delta z_{n}-\bar{\Delta z_{n}}\right)\left(\Delta z_{m}-\bar{\Delta z_{m}}\right)\left(\Delta z_{l}-\bar{\Delta z_{l}}\right)}=6\;b_{(ni}\;b_{mj}c_{l(ij))}+8\;c_{n(ij)}c_{m(jk)}c_{l(ik)}\;, \end{array}$$

where the round brackets indicate symmetrization with respect to the free indices, but not the dummy ones. The derivation details of Equation (14) are provided in Appendix A. The extra symmetrization brackets are placed around the indices *i* and *j* in the first term of the last expression in order to emphasize that it is only the symmetric part of $c_{lij}$ that contributes to the third cumulant $\kappa_{nml}$.

To obtain the cumulants (13) based on the initial state variable *z*, we resort to the Fokker–Planck equation (FPE) for the transition probability density $P(z,t|z_{0},t_{0})$ from the state $z_{0}$ at time $t_{0}$ to the state *z* at time *t* [2]:

(15) $$\dot{P}\left(z,t|z_{0},t_{0}\right)=\hat{L}\left(z\right)\;P\left(z,t|z_{0},t_{0}\right)={\left(\frac{1}{2}\;g_{ij}\;g_{kj}\;P_{,k}-\left(h_{i}-\frac{1}{2}\;g_{ij}\;g_{kj,k}\right)P\right)}_{,i}$$

with the initial condition

(16) $$P\left(z,t_{0}|z_{0},t_{0}\right)=\delta \left(z-z_{0}\right)\;.$$

The formal solution of the FPE reads

(17) $$P\left(z,t|z_{0},t_{0}\right)=e^{\hat{L}\left(z\right)\left(t-t_{0}\right)}\;\delta \left(z-z_{0}\right)\;.$$

Replacing the earlier time $t_{0}$ with *t* and the later time ts with t+Δt, we obtain the expectation value of an arbitrary state function $f(z^{\prime })$ at t+Δt given that, at time *t*, the system was in the state *z*:

(18) $$\begin{array}{c} \bar{f(z^{\prime })}=\int dz^{\prime }\;f\left(z^{\prime }\right)\;e^{\hat{L}\left(z^{\prime }\right)\;\Delta t}\;\delta \left(z^{\prime }-z\right)=\int dz^{\prime }\;\left(e^{{\hat{L}}^{†}\left(z^{\prime }\right)\;\Delta t}\;f\left(z^{\prime }\right)\right)\;\delta \left(z^{\prime }-z\right)=e^{{\hat{L}}^{†}\left(z\right)\;\Delta t}\;f\left(z\right)\;. \end{array}$$

Here, the adjoint Fokker–Planck operator is defined by

(19) $${\hat{L}}^{†}f=G_{ij}\;f_{,i,j}+H_{i}\;f_{,i}\;,$$

and the functions $H_{i}$ and $G_{ij}$ are introduced in Equation (8).

With $f\left(z^{\prime }\right)=e^{\zeta_{s}z_{s}^{\prime }}$, we obtain the characteristic function (12)

(20) $$\begin{array}{c} C\left(\zeta \right)=e^{-\zeta_{s}z_{s}}\;e^{{\hat{L}}^{†}\;\Delta t}\;e^{\zeta_{s}z_{s}}=1+e^{-\zeta_{s}z_{s}}\;\sum_{n=1}^{\infty }\frac{\Delta t^{n}}{n!}{\left({\hat{L}}^{†}\right)}^{n}e^{\zeta_{s}z_{s}}\;. \end{array}$$

Its natural logarithm is written with the help of the Taylor series

(21) $$\begin{array}{cc} \; & \ln C\left(\zeta \right)=\ln\left(1+S\right)=S-S^{2}/2+S^{3}/3-\ldots \;,\\ \; & S=e^{-\zeta_{s}z_{s}}\;\sum_{n=1}^{\infty }\frac{\Delta t^{n}}{n!}{\left({\hat{L}}^{†}\right)}^{n}e^{\zeta_{s}z_{s}}\;. \end{array}$$

Specifically, to the second order in Δt

(22) $$\begin{array}{cc} \ln C(\zeta ) & =\Delta t\;e^{-\zeta_{s}z_{s}}\;{\hat{L}}^{†}e^{\zeta_{s}z_{s}}+\frac{\Delta t^{2}}{2}\;\left(e^{-\zeta_{s}z_{s}}{\left({\hat{L}}^{†}\right)}^{2}e^{\zeta_{s}z_{s}}-e^{-2\zeta_{s}z_{s}}{\left({\hat{L}}^{†}e^{\zeta_{s}z_{s}}\right)}^{2}\right)+O\left(\Delta t^{3}\right)\\ \; & =\Delta t\;\left(G_{ij}\;\zeta_{i}\zeta_{j}+H_{i}\;\zeta_{i}\right)+\frac{\Delta t^{2}}{2}\;\left({\hat{L}}^{†}\left(G_{ij}\;\zeta_{i}\zeta_{j}+H_{i}\;\zeta_{i}\right)+2G_{kl}\;\left(G_{ij,k}\zeta_{i}\zeta_{j}+H_{i,k}\zeta_{i}\right)\;\zeta_{l}\right)\\ \; & +O(\Delta t^{3})\;. \end{array}$$

Here, the terms that multiply Δt and $\Delta t^{2}$ were grouped together, and then the identity

(23) $${\hat{L}}^{†}e^{\zeta_{s}z_{s}}=\left(G_{ij}\zeta_{i}\zeta_{j}+H_{i}\zeta_{i}\right)\;e^{\zeta_{s}z_{s}}$$

was used together with the “product rule” valid for arbitrary state functions ϕ(z) and χ(z):

(24) $${\hat{L}}^{†}\left(\phi \chi \right)=\phi {\hat{L}}^{†}\chi +\chi {\hat{L}}^{†}\phi +2G_{ij}\phi_{,i}\chi_{,j}\;.$$

Differentiating Equation (22), we obtain the first three cumulants:

(25) $$\begin{array}{cc} \; & \kappa_{n}=\Delta t\;H_{n}+\frac{\Delta t^{2}}{2}\;{\hat{L}}^{†}H_{n}+O\left(\Delta t^{3}\right)\;,\\ \; & \kappa_{nm}=2G_{nm}\;\Delta t+\left(2\;G_{i(n}\;H_{m),i}+{\hat{L}}^{†}G_{nm}\right)\;\Delta t^{2}\;,\\ \; & \kappa_{nml}=6\;\Delta t^{2}\;G_{i(n}\;G_{ml),i}+O\left(\Delta t^{3}\right)\;. \end{array}$$

The first line immediately gives an expression for the coefficient $a_{n}$ from Equation (5) according to the first Equation (14). We now need to solve the remaining two Equations in (14) with the cumulants $\kappa_{nm}$ and $\kappa_{nml}$ from Equation (25) to find the coefficients $b_{nm}$ and $c_{nml}$. We look for them in the form of expansions in the time step:

(26) $$\begin{array}{cc} \; & b_{nm}=\alpha_{nm}\;\sqrt{\Delta t}+\beta_{nm}\;\Delta t^{3/2}+O\left(\Delta t^{5/2}\right)\;,\\ \; & c_{nml}=\Delta t\;\sigma_{nml}+O\left(\Delta t^{2}\right)\;. \end{array}$$

Substitution of these expansions into Equation (14) gives the second and the third cumulants in terms of the yet-unknown coefficients $\alpha_{nm}$, $\beta_{nm}$, and $\sigma_{nml}$:

(27) $$\begin{array}{c} \kappa_{nm}=\alpha_{ni}\;\alpha_{mi}\;\Delta t+2\;\Delta t^{2}\;\left(\alpha_{(ni}\;\beta_{mi)}+\sigma_{n(ij)}\;\sigma_{m(ij)}\right)+O\left(\Delta t^{3}\right),\\ \kappa_{nml}=6\;\Delta t^{2}\;\alpha_{(ni}\;\alpha_{mj}\;\sigma_{l(ij))}+O\left(\Delta t^{3}\right)\;. \end{array}$$

Comparing the term proportional to Δt in $\kappa_{nm}$ with the second Equation (25), we find that $\alpha_{ni}\alpha_{mi}=2G_{nm}=g_{ni}g_{mi}$. Since this equality must hold for an arbitrary function $g_{nm}\left(z\right)$, we can identify

(28) $$\alpha_{nm}=g_{nm}\;.$$

Next, we go on to the calculation of $\sigma_{nml}$ based on the third cumulant in Equation (25), which is rewritten as

(29) $$\kappa_{nml}=3\;\Delta t^{2}\;g_{(nj}\;g_{mk}\;g_{lk,i}\;g_{ij)}+O\left(\Delta t^{3}\right)\;.$$

Here, we used the fact that $G_{in}=G_{ni}=\frac{1}{2}\;g_{nj}\;g_{ij}$ and $G_{ml,i}=\frac{1}{2}\left(g_{mk,i}g_{lk}+g_{mk}g_{lk,i}\right)=g_{(mk}g_{lk),i}$.

Due to the symmetrization, we can interchange the indices *n* and *m* in Equation (29). Further, we interchange the dummy indices *j* and *k* and write

(30) $$\kappa_{nml}=3\;\Delta t^{2}\;g_{(nj}\;g_{mk}\;g_{lj,i}\;g_{ik)}+O\left(\Delta t^{3}\right)\;.$$

On the other hand, the second Equation (27) and Equation (28) give

(31) $$\kappa_{nml}=6\;\Delta t^{2}\;g_{(nj}\;g_{mk}\;\sigma_{l(jk))}+O\left(\Delta t^{3}\right)\;.$$

A comparison of Equation (31) with the arithmetic average of Equations (29) and (30) allows one to identify

(32) $$\sigma_{l(jk)}=\frac{1}{2}\;g_{l(j,i}\;g_{ik)}\;,\;\;\sigma_{ijk}=\frac{1}{2}\;g_{ij,l}\;g_{lk}\;,$$

as stated in the third Equation (7) and Equation (8).

We substitute the expressions for $\alpha_{nm}$ and $\sigma_{nml}$ into the first Equation (27) and compare the terms that multiply $\Delta t^{2}$ with the respective terms in the second Equation (25). Based on the product rule (24), we can write

(33) $$\begin{array}{c} {\hat{L}}^{†}G_{nm}=\frac{1}{2}\;{\hat{L}}^{†}\left(g_{ni}g_{mi}\right)=g_{(ni}\;{\hat{L}}^{†}g_{mi)}+\frac{1}{2}\;g_{kj}\;g_{lj}\;g_{ni,k}\;g_{mi,l}=g_{(ni}\;{\hat{L}}^{†}g_{mi)}+2\;\sigma_{nij}\;\sigma_{mij}\;, \end{array}$$

where the previously obtained expression for $\sigma_{ijk}$ is used in the last equality. The first term in the brackets of the second expression (25) is $G_{i(n}H_{m),i}=\frac{1}{2}\;g_{(ij}g_{nj}H_{m,i)}$. Hence,

(34) $$\begin{array}{c} g_{(ni}\beta_{mi)}=\frac{1}{2}\left(g_{(ni}g_{ji}H_{m,j)}+g_{(ni}{\hat{L}}^{†}g_{mi)}\right)+\sigma_{nij}\;\sigma_{mij}-\sigma_{n(ij)}\;\sigma_{m(ij)} \end{array}$$

To deal with the difference of the σ’s in the second line, we express these coefficients as a sum of symmetric and antisymmetric parts, $\sigma_{nij}=\sigma_{n(ij)}+\sigma_{n[ij]}$, and note that $\sigma_{n(ij)}\sigma_{n[ij]}=0$. Then, the second line in Equation (34) is just

(35) $$\begin{array}{cc} \sigma_{nij}\sigma_{mij} & -\sigma_{n(ij)}\sigma_{m(ij)}=\sigma_{n[ij]}\;\sigma_{m[ij]}=\delta_{nk}\;\sigma_{k[jl]}\;\sigma_{m[jl]}=g_{(ni}{\left(g^{-1}\right)}_{ik}\sigma_{k[jl]}\sigma_{m[jl])}\;. \end{array}$$

The symmetrization brackets are placed around the subscripts in the last step to emphasize that the expression obtained is symmetric with respect to the free indices *n* and *m*, as is obvious from the right-hand side of Equation (35). Substitution of this result into Equation (34) finally gives

(36) $$\beta_{mi}=\frac{1}{2}\;\left(H_{m,j}g_{ji}+{\hat{L}}^{†}g_{mi}\right)+\sigma_{m[jl]}\;\sigma_{k[jl]}\;{\left(g^{-1}\right)}_{ik}\;,$$

thereby completing the derivation of the coefficients (7) in the propagation rule (5).

## 4. Case Study: Transport Induced by Periodic Spatial Modulation of Temperature

Ratchet effect refers to transport in a noisy system, whose parameters are periodically modulated around the average values in such a way that transport in the absence of this modulation is impossible [20]. Usually, the parameter modulation occurs in time [20]; however, the above definition may as well be applied to the situations in which the modulation happens in space, as demonstrated by Büttiker [21] in the earliest example of a ratchet effect induced by spatial modulation of temperature.

To evaluate the improvements introduced by the present scheme over the first-order methods, we compare it with the Milstein algorithm, which arises as the leading-order truncation of our formulation. In particular, we wish to examine the role of higher-order contributions—specifically, the terms of order $\Delta t^{3/2}$ and $\Delta t^{2}$—that are absent in the Milstein method. As a testing ground, we choose the Büttiker ratchet system, which permits comparison of not only the static properties via the equilibrium probability distribution, but also the dynamical features, captured by the mean particle velocity.

**Figure 1:**
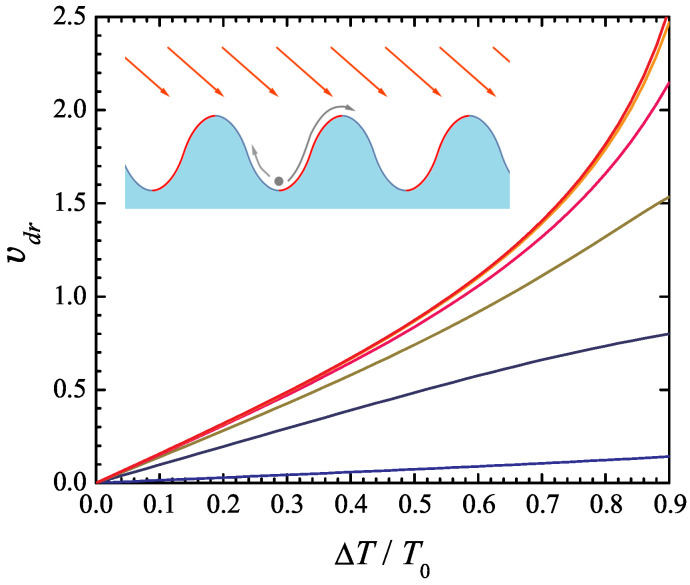
Drift velocity of an overdamped Brownian particle in Büttiker’s ratchet model (37), (38) as a function of the temperature modulation amplitude ΔT normalized to the average temperature $T_{0}$. The model parameters $U_{0}$, *a*, and γ are set to 1. The six curves represent different values of $T_{0}=$ 20, 5, 2, 1, 0.5, and 0.2 (from top to bottom). The inset illustrates the physical interpretation of the system, where a periodic potential and spatial temperature variations induce directed transport.

Let us consider the Büttiker’s ratchet model [21] of an overdamped Brownian particle in a periodic potential U(x)=U(x+a) with periodicity *a* in a non-uniform temperature field T(x)=T(x+a) with the same spatial periodicity as the potential. The LE reads

(37) $$\gamma \dot{x}=-\frac{dU}{dx}+\sqrt{2\gamma T(x)}\;\xi \left(t\right)\;,$$

where γ is the damping coefficient and T(x) is the position-dependent temperature. For definiteness, we assume the potential and the temperature to be given by

(38) $$U\left(x\right)=-\frac{U_{0}}{2}\;\cos\left(\frac{2\pi x}{a}\right)\;,\;\;T\left(x\right)=T_{0}+\Delta T\;\sin\left(\frac{2\pi x}{a}\right)\;,$$

where $U_{0}$ is the potential corrugation depth, $T_{0}$ is the average temperature, and ΔT is the temperature modulation amplitude. A simple way of thinking about this model is to consider a particle in a gravity field moving in a periodic terrain, see inset in Figure 1. When light is incident on this landscape at an angle, it induces a non-uniform heating effect, with the illuminated regions becoming hotter than the shaded areas.

In the absence of spatial temperature variations, the model does not exhibit net motion. Likewise, periodic temperature variations alone, without a corresponding periodic potential, do not induce a net drift. However, when both the temperature and potential vary periodically in space and are phase-shifted relative to each other, the probabilities of a particle transitioning from one potential well to an adjacent one—either to the left or right—are generally unequal. Specifically, the probability of transition over the “hotter” side of the potential well is greater than that over the “colder” side. This asymmetry leads to a net transport of the particle. It has recently been suggested [22] that this effect can drive a semiconductor thermoelectric generator.

An analytical expression for the drift velocity can be developed following the treatment of Ref. [21]. Namely, the FPE for the probability density P(x,t) to find the particle near the position *x* at time *t*,

(39) $$\dot{P}\left(x,t\right)=\frac{1}{\gamma }\;\frac{\partial }{\partial x}\left(T\left(x\right)\;\frac{\partial P}{\partial x}+\left(\frac{dU}{dx}+\frac{1}{2}\;\frac{dT}{dx}\right)\;P\right)\;,$$

has the form of a continuity equation, $\dot{P}=-\partial J/\partial x$, where J(x,t) is the probability current. We look for the stationary solution of the FPE (39) that respects the periodic boundary conditions and is normalized to 1 within one period:

(40) $$P_{st}\left(x+a\right)=P_{st}\left(x\right)\;,\;\;\int_{0}^{a}dx\;P_{st}\left(x\right)=1\;.$$

The probability current is constant and equals

(41) $$J_{st}=-\frac{1}{\gamma }\;\left(T\left(x\right)\;\frac{dP_{st}}{dx}+\left(\frac{dU}{dx}+\frac{1}{2}\;\frac{dT}{dx}\right)\;P_{st}\right)\;.$$

By solving Equation (41) with the periodicity conditions (38), we first express $P_{st}\left(x\right)$ in terms of the probability current as

(42) $$P_{st}\left(x\right)=\frac{\gamma J_{st}\;\int_{x}^{x+a}\frac{dy}{\sqrt{T(y)}}\;e^{\psi (y)-\psi (x)}}{\left(1-e^{\psi (a)}\right)\;\sqrt{T(x)}}\;,\;\;\psi \left(x\right)=\int_{0}^{x}\frac{dy}{T(y)}\;\frac{dU(y)}{dy}\;.$$

Imposing the normalization condition (40) and noting that the probability current is related to the drift velocity of the particle by $J_{st}=v_{dr}/a$, we obtain the drift velocity as [21]

(43) $$v_{dr}=\frac{a\;(1-e^{\psi (a)})}{\gamma \;\int_{0}^{a}\frac{dx}{\sqrt{T(x)}}\;\int_{x}^{x+a}\frac{dy}{\sqrt{T(y)}}\;e^{\psi (y)-\psi (x)}}\;.$$

In the numerical simulations, the parameters *a*, $U_{0}$, and γ are set to 1, thereby fixing the units of length, energy, and time. The drift velocity (43) vs. the ratio of the temperature modulation amplitude ΔT to the average temperature $T_{0}$ is shown in Figure 1 for $T_{0}\;=\;20,5,2,1,0.5$, and 0.2 (from top to bottom). As might be expected, the drift velocity increases with the temperature modulation amplitude, as well as with the average temperature $T_{0}$ at a fixed ratio $\Delta T/T_{0}$. Somewhat less obvious is the fact that the drift velocity vs. $\Delta T/T_{0}$ curve becomes less sensitive to the average temperature $T_{0}$ with increasing its value. Indeed, the curves $v_{dr}$ vs. $\Delta T/T_{0}$ obtained for $T_{0}=5$ and $T_{0}=20$ differ very little; further increase in $T_{0}$ above the value 20 does not result in its noticeable change.

The stochastic trajectories of the Brownian particle (38) were simulated according to the algorithm from Section 2, which, in the one-dimensional case, simplifies to

(44) $$\begin{array}{c} x\left(t+\Delta t\right)=x\left(t\right)+a+b\;\Gamma +c\left(\Gamma^{2}-1\right)\;,\\ a=\Delta tH+\frac{\Delta t^{2}}{2}\left(G\frac{d^{2}H}{dx^{2}}+H\frac{dH}{dx}\right)\;,\;\;b=g\sqrt{\Delta t}+\frac{\Delta t^{3/2}}{2}\left(g\frac{dH}{dx}+G\frac{d^{2}g}{dx^{2}}+H\frac{dg}{dx}\right)\;,\\ c=\frac{\Delta t}{2}\;\frac{dG}{dx}\;,\;\;g\left(x\right)=\sqrt{\frac{2T(x)}{\gamma }}\;,\;\;G\left(x\right)=\frac{1}{2}g^{2}\left(x\right)\;,\;\;H\left(x\right)=-\frac{1}{\gamma }\;\frac{dU}{dx}+\frac{1}{2}\;\frac{dG}{dx}\;. \end{array}$$

If only the first-order term is kept in the expression for *a* and only the term of the order of $\sqrt{\Delta t}$ is kept in the expression for *b*, the scheme (44) becomes identical with the standard Milstein method [10].

The simulations were performed according to the algorithm (44) and following the Milstein scheme at several average temperatures $T_{0}=0.2,1$, and 5. For all values of $T_{0}$, the temperature amplitude was kept at $\Delta T=T_{0}/2$. To determine the drift velocity $v_{simul}=x\left(t_{max}\right)/t_{max}$ from the simulations, the particle trajectory was generated over a long time $t_{max}=10^{5}$ with the initial condition x(0)=0. The statistical uncertainty of the simulation results was below 1% in all cases.

**Figure 2:**
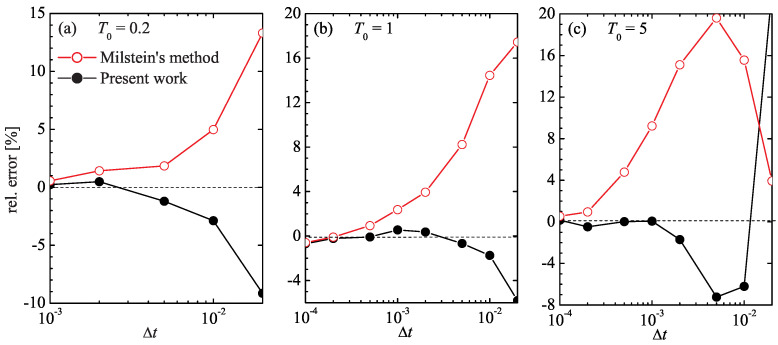
Relative error, $\frac{v_{simul}-v_{dr}}{v_{dr}}\;\times \;100\;\%$, of the drift velocity obtained from the numerical simulation of Equation (37) using the Milstein method (red open circles) and the method of the present work (black filled circles) for different values of the simulation time step Δt.

Shown in Figure 2 is the relative deviation of the average velocity $v_{simul}$ from the exact value (43), $(v_{simul}/v_{dr}-1)\times 100\%$, for both simulation algorithms at different time-step values Δt. It is seen that, at low average temperature $T_{0}=0.2$, both schemes exhibit about the same accuracy, even though the Milstein scheme overestimates the drift velocity, while the algorithm (43) underestimates it by a slightly smaller amount; for example, at Δt=0.01, the error of the Milstein method is close to 5%, while the scheme (43) has an error of about 3%.

The discrepancy of the two methods becomes more evident at higher temperatures. At $T_{0}=1$, the scheme (43) achieves a 1% accuracy at $\Delta t=5\times 10^{-3}$, whereas the Milstein approach requires $\Delta t=5\times 10^{-4}$. Likewise, at $T_{0}=5$, the scheme (43) achieves this accuracy at $\Delta t=10^{-3}$, whereas the Milstein procedure requires a time step ten times as small.

The similarity in the mean velocity calculation by the two methods at low temperature, Figure 2a, does not necessarily imply that they are equally accurate in this regime. Indeed, in the deterministic limit $T_{0}\to 0$, our scheme (43) reduces to a second-order Taylor expansion of $x\left(t+\Delta t\right)=x\left(t\right)+\dot{x}\left(t\right)\;\Delta t+\ddot{x}\left(t\right)\;\Delta t^{2}/2$ with velocity $\dot{x}=-\gamma^{-1}\;dU/dx$ and acceleration $\ddot{x}=-\gamma^{-1}\;\dot{x}\;d^{2}U/dx^{2}$, whereas the Milstein scheme only contains the velocity term. Nevertheless, although the Milstein scheme is only first-order accurate, it still correctly yields the zero drift velocity at zero temperature $T_{0}$. One can expect that at low but finite temperatures, even a rudimentary method can capture the near-zero drift velocity with seemingly good accuracy. To properly differentiate the performance of integration schemes in this regime, one would need to examine a quantity more sensitive than $v_{dr}$.

**Figure 3:**
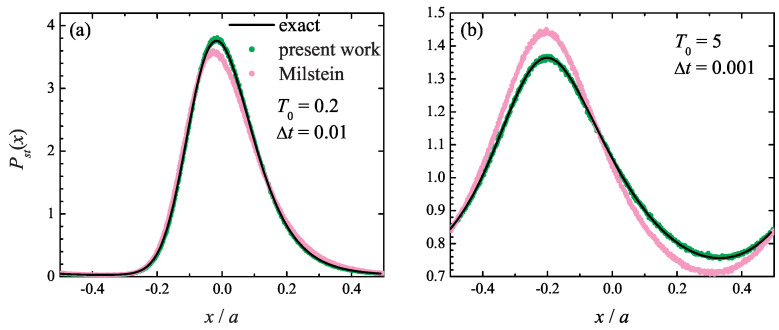
Particle steady-state periodic probability distribution in the Büttiker ratchet model at $\Delta T=T_{0}/2$ at (**a**) $T_{0}=0.2$ and integration time step Δt=0.01 and (**b**) $T_{0}=5$ and Δt=0.001. Solid line: exact curve (44); green symbols: simulation results obtained with the present method; magenta symbols: simulation results using the Milstein scheme.

Such a quantity may be the equilibrium probability distribution $P_{st}\left(x\right)$ itself, given by Equation (42). It is shown in Figure 3 at (a) $T_{0}=0.2$ and (b) $T_{0}=5$, where the exact distribution (42) is compared with the one found from the simulations based on the present method (5)–(7) and the Milstein algorithm. It is seen that the Milstein integration method yields quantitatively inaccurate steady-state probability distribution $P_{st}\left(x\right)$ at both temperatures, even though its estimate of the drift velocity is close to the correct value. At the same time, the numerical results obtained with the present method (5)–(7) are in excellent agreement with the theoretical curve, which highlights the importance of the higher-order terms in the stochastic Taylor expansion (5).

## 5. Concluding Remarks

A numerical method is worked out for generating stochastic trajectories that preserve the cumulants of the state variable up to the second order in the time step. The derivation presented here applies to the white noise-driven systems of arbitrary dimensionality *N*. The accuracy of this approach in computing observables is comparable to the accuracy of the second-order stochastic Taylor expansion-based methods [4,5]. The advantage of the present approach lies in the fact that it reduces computational complexity, because it requires a single set of *N*-independent Gaussian random variables. In contrast, the stochastic Taylor expansion-based methods require random numbers of the order $N^{4}$ with specific correlations among them (see, e.g., Equations (10)–(15) of Ref. [8]). Their generation is a non-trivial task, especially at large *N*.

In terms of the idea used in the derivation, the closest multidimensional algorithm published in the literature is by Cao and Pope (abbreviated as CP; see Section 2.4 of [18]), as both methods provide a second-order weak integration scheme for the Langevin Equation (3), and both are based on matching the average properties of the updated system’s state using the associated Fokker–Planck Equation (15). While the conceptual foundation is similar, there are several important distinctions between the present algorithm and the CP method. First, the CP algorithm is a midpoint scheme: it requires one to evaluate the state variable at time $t_{n}+\Delta t/2$ before computing the full time-step update $z\left(t_{n}\right)\to z\left(t_{n+1}\right)$. In contrast, the numerical scheme (5)–(7) performs this time-step propagation directly. Second, the CP scheme uses three *N*-dimensional sets of uncorrelated Gaussian random variables; the present method requires only one such set. Finally, the CP scheme is formulated for the special case in which the noise-coupling matrix $g_{ij}\left(z\right)$ reduces to a scalar function common to all components $z_{n}$ of the state vector *z*, whereas the scheme developed here allows for a fully general, position-dependent noise matrix.

To improve the performance speed of the scheme (5), one may be tempted to replace Gaussian random numbers $\Gamma_{i}$ with a different type of random numbers that can be generated more quickly [8,23]. However, there is strong evidence [24] that using non-Gaussian random variables can worsen the accuracy of the method. Indeed, the properties of the Gaussian numbers were explicitly used in deriving the cumulant expressions (14); attempting to replace $\Gamma_{i}$ with non-Gaussian random variables may require a major modification of the derivation of the propagation rule (5), and thus to a major modification of this rule itself.

If the terms of order higher than $\Delta t^{1}$ are neglected in the scheme (5)–(7), it reduces to the general multidimensional form of the Milstein method [10]. For this reason, the update rule (5)–(7) may be regarded as a second-order explicit weak Milstein method. It is logical to focus the future research on exploring the higher-order corrections to the scheme (5), (7). When doing this, adding terms of the higher order in Δt to the coefficient $a_{n}$, $b_{nm}$, and $c_{nml}$ may not necessarily lead to better accuracy of the algorithm. The reason is that increasing the order of the algorithm results in the emergence of the higher-order cumulants, as can be shown based on Equations (20) and (21). In particular, the leading term in the fourth cumulant is of the order of $\Delta t^{3}$; hence, if one wishes to extend the order of the scheme (5), (7) to $\Delta t^{3}$, one would need to impose an extra condition that the fourth cumulant $\kappa_{nmlk}$ is correctly reproduced by the updated state variable $z^{\prime }$. This, in turn, implies that an extra term $d_{nmlk}\;\Gamma_{m}\Gamma_{l}\Gamma_{k}$ should be added to the stochastic Taylor expansion (5) with the unknown parameters $d_{nmlk}$. Thus, going to the higher order in Δt will result in higher computational complexity, but may potentially be beneficial for the accuracy of the method.

## Data Availability

The data presented in this study are available on request from the corresponding author.

## Appendix A. Properties of Gaussian Random Numbers

To derive Equation (14), Equation (5) was used together with the average value of the product of Gaussian random variables (6):

$$\bar{\Gamma_{i_{1}}\ldots \Gamma_{i_{2k+1}}}=0\;\;,\;\;\;\bar{\Gamma_{i_{1}}\ldots \Gamma_{i_{2k}}}=\frac{(2k)!}{2^{k}\;k!}\;\delta_{(i_{1}i_{2}}\ldots \delta_{i_{2k-1}i_{2k})}\;,$$

see, e.g., Section 2.3.3 of Ref. [2]. The last line represents a sum over all different combinations of Kronecker’s deltas; for example, $\bar{\Gamma_{i}\Gamma_{j}\Gamma_{k}\Gamma_{l}}=\delta_{ij}\delta_{kl}+\delta_{ik}\delta_{jl}+\delta_{il}\delta_{kj}$.

More generally, the mean value of the product

$$\bar{\Gamma_{i_{1}}\ldots \Gamma_{i_{2k}}\left(\Gamma_{j_{1}}\Gamma_{j_{2}}-\delta_{j_{1}j_{2}}\right)\ldots \left(\Gamma_{j_{2n-1}}\Gamma_{j_{2n}}-\delta_{j_{2n-1}j_{2n}}\right)}$$

is a sum of different products of Kronecker deltas with all combinations of the pairs of indices from $i_{1},\ldots ,i_{2k}$, $j_{1},\ldots ,j_{2n}$, excluding the ones that contain the deltas found in the brackets, i.e., $\delta_{j_{1}j_{2}}$, …, $\delta_{j_{2n-1}j_{2n}}$. This can be proven by induction. Indeed, this statement is obviously true for n=1 and arbitrary *k*, as

$$\bar{\Gamma_{i_{1}}\ldots \Gamma_{i_{2k}}\;\left(\Gamma_{j_{1}}\Gamma_{j_{2}}-\delta_{j_{1}j_{2}}\right)}=\frac{2(k+1)!}{2^{k+1}\left(k+1\right)!}\;\delta_{(i_{1}i_{2}}\ldots \delta_{i_{2k-1}i_{2k}}\;\delta_{j_{1}j_{2})}-\frac{2k!}{2^{k}k!}\;\delta_{(i_{1}i_{2}}\ldots \delta_{i_{2k-1}i_{2k})}\;\delta_{j_{1}j_{2}}$$

Assuming that it holds for some *n* and arbitrary *k*, we write the expectation value for n+1 as a difference:

$$\begin{array}{cc} \; & \bar{\Gamma_{i_{1}}\ldots \Gamma_{i_{2k}}\Gamma_{j_{2n+1}}\Gamma_{j_{2n+2}}\left(\Gamma_{j_{1}}\Gamma_{j_{2}}-\delta_{j_{1}j_{2}}\right)\ldots \left(\Gamma_{j_{2n-1}}\Gamma_{j_{2n}}-\delta_{j_{2n-1}j_{2n}}\right)}\\ \; & -\delta_{j_{2n+1}j_{2n+2}}\bar{\Gamma_{i_{1}}\ldots \Gamma_{i_{2k}}\left(\Gamma_{j_{1}}\Gamma_{j_{2}}-\delta_{j_{1}j_{2}}\right)\ldots \left(\Gamma_{j_{2n-1}}\Gamma_{j_{2n}}-\delta_{j_{2n-1}j_{2n}}\right)}\;. \end{array}$$

By the inductive assumption, the first average represents the sum of products of deltas $\delta_{i_{1}i_{2}}$, $\delta_{i_{1}i_{3}}$, …, $\delta_{j_{2n+1}j_{2n+2}}$, from which the ones that contain $\delta_{j_{1}j_{2}}$, …, $\delta_{j_{2n-1}j_{2n}}$ are excluded. The second term represents the subset of products of deltas found in the first average that contain $\delta_{j_{2n+1}j_{2n+2}}$. The difference between the two averages is, therefore, the sum of products of all deltas, from which $\delta_{j_{1}j_{2}}$, …, $\delta_{j_{2n+1}j_{2n+2}}$ are excluded.

With these properties, we immediately obtain the first cumulant in Equation (14). The second cumulant is

$$\begin{array}{cc} \kappa_{nm} & =b_{ni}b_{mi}\;\bar{\Gamma_{i}\Gamma_{j}}+c_{nij}c_{mkl}\;\bar{\left(\Gamma_{i}\Gamma_{j}-\delta_{ij}\right)\left(\Gamma_{k}\Gamma_{l}-\delta_{kl}\right)}\\ \; & =b_{ni}b_{mi}\;\delta_{ij}+c_{nij}c_{mkl}\left(\delta_{ik}\delta_{jl}+\delta_{il}\delta_{jk}\right)=b_{ni}b_{mi}+c_{nij}\;\left(c_{mij}+c_{mji}\right)=b_{ni}b_{mi}+2c_{nij}c_{m(ij)}\;. \end{array}$$

In the last line, $c_{nij}=c_{n(ij)}+c_{n[ij]}$ can be replaced with just $c_{n(ij)}$, because $c_{n[ij]}c_{n(ij)}=0$. The third cumulant in Equation (14) is derived in a similar manner using the identities

$$\begin{array}{cc} \; & \bar{\Gamma_{i}\Gamma_{j}\left(\Gamma_{k}\Gamma_{l}-\delta_{kl}\right)}=\delta_{ik}\delta_{jl}+\delta_{il}\delta_{jk}\;,\\ \; & \bar{\left(\Gamma_{i}\Gamma_{j}-\delta_{ij}\right)\left(\Gamma_{k}\Gamma_{l}-\delta_{kl}\right)\left(\Gamma_{m}\Gamma_{n}-\delta_{nm}\right)}\\ \; & \;\;\;\;=\delta_{ik}\delta_{lm}\delta_{nj}+\delta_{ik}\delta_{ln}\delta_{mj}+\delta_{il}\delta_{km}\delta_{nj}+\delta_{il}\delta_{kn}\delta_{mj}\\ \; & \;\;\;\;+\delta_{im}\delta_{jk}\delta_{ln}+\delta_{im}\delta_{jl}\delta_{kn}+\delta_{in}\delta_{jk}\delta_{lm}+\delta_{in}\delta_{jl}\delta_{km}\;. \end{array}$$
